# Metabolic biomarkers mediate allergic rhinitis via circulating inflammatory proteins: Evidence from a Mendelian randomization study

**DOI:** 10.1016/j.bjorl.2025.101658

**Published:** 2025-06-25

**Authors:** Xiang Cao, Boyang Zhang, Wei Wang, Zijiao Xu, Zhixin Jiang

**Affiliations:** a Jiangsu University, Affiliated People’s Hospital, Department of Ophthalmology, Zhenjiang, Jiangsu, China; b Zhenjiang Kangfu Eye Hospital, Zhenjiang, Jiangsu, China; c Clinical College of Ophthalmology, Tianjin Medical University, Tianjin, China; d Tianjin Eye Hospital, Nankai University Affiliated Eye Hospital, Clinical College of Ophthalmology, Tianjin Medical University, Tianjin Eye Institute, Tianjin Key Laboratory of Ophthalmology and Visual Science, Tianjin, China; e Shanxi Medical University, Shanxi, China

**Keywords:** Allergic rhinitis, Mendelian randomization, Metabolic biomarkers, Circulating inflammatory proteins

## Abstract

**Objective:**

Allergic Rhinitis (AR), a prevalent allergic condition, markedly diminishes quality of life and increases public health burdens. This study investigated the mediating roles of Metabolic Biomarkers (MBs) in the associations between Circulating Inflammatory Proteins (CIPs) and AR, aiming to uncover novel therapeutic targets.

**Methods:**

Utilizing Mendelian Randomization (MR), we leveraged genetic variants as instrumental variables to delineate causal relationships between CIPs and AR. Data were extracted from comprehensive Genome-Wide Association Studies (GWAS). A two-sample MR approach was employed to assess both the direct and mediated effects of CIPs on AR through MBs. Inverse-Variance Weighting (IVW) served as the main analytical method, supplemented by rigorous sensitivity analyses to confirm the robustness of the findings.

**Results:**

Our analysis revealed that specific CIPs like C-C motif Chemokine-19 (CCL19), Notch-like Epidermal growth factor-related Receptor (DNER), and Interleukin-6 (IL-6) significantly elevate the risk of AR, whereas Interleukin-10 (IL-10) exhibit protective properties. Key MBs, notably Adenosine 5'-Monophosphate (AMP) to urate ratio, plasma lactate, and N-acetylputrescine, played critical roles in mediating these relationships, indicating their potential as therapeutic targets.

**Conclusion:**

This investigation highlights the pivotal role of MBs in modulating the influence of CIPs on AR. The insights gained from this study not only deepen our understanding of AR pathophysiology but also open new avenues for metabolic intervention in its treatment.

**Level of evidence:**

In terms of level of evidence, MR studies are second only to randomized controlled trials.

## Highlights

• Used genetic data to clarify causes of AR, reducing confounding factors.
• Genetic evidence supports IL-6, CCL19, and DNER as risk factors for AR.
• IL-10 exhibits a protective effect against AR.
• Key MBs (AMP-to-urate ratio, lactate, N-acetylputrescine) mediate AR risk.

## Introduction

Allergic Rhinitis (AR) is the most prevalent form of type I allergy in industrialized countries^1^ and represents a common chronic inflammatory condition of the nasal airways.^2^ It is characterized by symptoms such as sneezing, nasal congestion, itching, and watery discharge.^3^ The global prevalence of AR has been rising, affecting individuals across all age groups.^4^ AR can significantly impair quality of life by causing sleep disturbances, fatigue, reduced concentration, and decreased productivity.^5^, ^6^ Moreover, it frequently coexists with other allergic disorders, such as asthma and eczema, contributing to the concept of the “allergic march”.^7^, ^8^ The pathophysiology of AR involves a complex interplay between genetic predisposition and environmental factors. AR occurs when the immune system overreacts to harmless allergens like pollen, dust mites, pet dander, or mold spores, triggering an immune response that leads to the release of histamines and other inflammatory mediators.^9^, ^10^

Circulating Inflammatory Proteins (CIPs) are soluble mediators that play a key role in various physiological and pathological processes.^11^, ^12^ These proteins are secreted by cells and tissues in response to immune stimuli, such as infection or injury, and are transported via the bloodstream, where they regulate immune responses, tissue repair, and disease progression.^13^ Recent research has underscored the critical role of CIPs and Metabolic Biomarkers (MBs) in allergic diseases.^9^, ^14^ For example, omega-3 fatty acids and their metabolites have been identified as potential anti-inflammatory agents with promising effects on conditions characterized by excessive inflammation.^15^ Similarly, Th2 cell involvement is crucial in the development and progression of allergic diseases.^16^, ^17^ A notable study by Bai et al. demonstrated that regulating the NLRP3-IL-17 signaling axis can alleviate symptoms in AR patients.^18^ Therefore, further investigation into the causal relationships between CIPs, their MBs, and AR could provide valuable insights into the disease’s progression and identify potential therapeutic targets.

Previous studies may have been confounded by unrecognized variables or reverse causality, making it difficult to establish definitive causal links.^19^ Traditional observational analyses often rely on statistical adjustments to control for confounding, but residual confounding from unmeasured factors remains a challenge. Furthermore, observational associations can be biased due to reverse causality, where the outcome influences the exposure rather than the other way around.^19^, ^20^ Mendelian Randomization (MR) is an analytical approach that leverages genetic variation from observational data to infer the causal effects of an exposure on an outcome. Since alleles are randomly assigned during meiosis, MR reduces bias from confounding and reverse causality, offering stronger evidence for causal inference.^21^

In this study, we are the first to apply mediated MR methods to investigate the role of 1,400 MBs in the relationship between 91 CIPs and AR. By integrating Genome-Wide Association Study (GWAS) data with relevant biomarkers, we aim to unravel the intricate connections between genetic susceptibility, inflammatory responses, and AR. Our findings are expected to enhance the understanding of these widespread allergic conditions and guide the development of targeted, effective interventions.

## Methods

### MR design

**Fig. 1:**
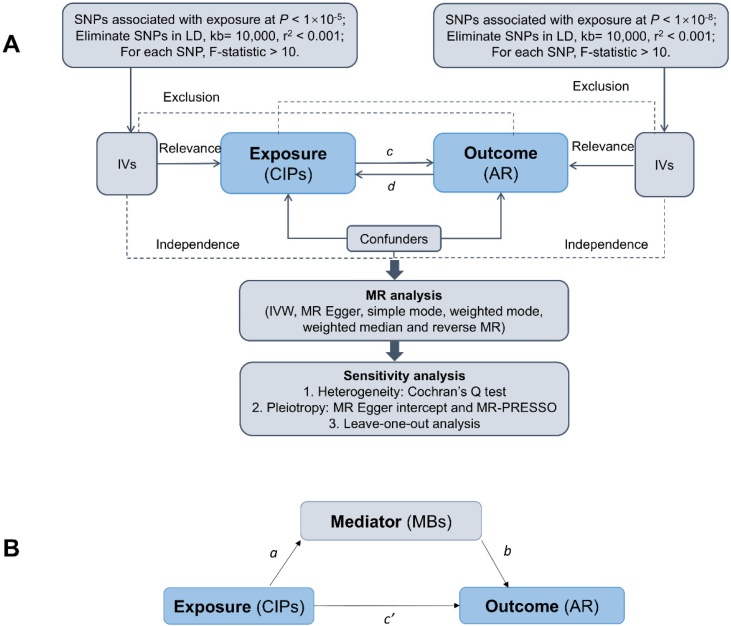
Schematic diagram of this study. (A) Flowchart of the bidirectional MR Study. The total effect c was analyzed using genetically predicted CIPs as the exposure and AR as the outcome. Additionally, the total effect d was also analyzed using genetically predicted AR as the exposure and CIPs as the outcome. (B) The total effect was further broken down into (I) the indirect effect, which was assessed using a two-step approach involving the effects of CIPs on MBs (a) and the effects of MBs on AR (b), as well as the product method (a × b); and (II) the direct effect, calculated as c′ = c − a × b. The proportion mediated was determined by dividing the indirect effect by the total effect. CIPs, Circulating Inflammatory Proteins; AR, Allergic Rhinitis; LD, Linkage disequilibrium; SNPs, Single nucleotide polymorphisms; MR, Mendelian randomization; MBs, Metabolic Biomarkers.

In this study, we employed mediation MR to investigate the mediating role of MBs in the relationship between CIPs and AR risk. Single Nucleotide Polymorphisms (SNPs) were defined as Instrumental Variables (IVs). MR analysis is based on three core assumptions: 1) IVs must be strongly associated with the exposure; 2) IVs must not be associated with any confounding factors; and 3) IVs must influence the outcome only through the exposure.^22^ The results of the study were visually represented using forest plots, funnel plots, scatter plots, and leave-one-out plots. The study design is illustrated in Fig. 1. All statistical analyses were conducted using the “TwoSampleMR” package (version 0.5.6) and the “MRPRESSO” package (version 1.0) in *R* software (version 4.3.2).

### Data sources and selection of genetic instruments

Genetic associations for AR were obtained from the FinnGen consortium,^23^ which included 12,240 cases and 392,069 controls. Additional details can be accessed at (https://www.finngen.fi/en/access_results). The data for 91 CIPs were sourced from a GWAS summary dataset comprising 14,824 individuals of European ancestry.^11^ The plasma metabolome dataset was derived from the Canadian Longitudinal Study of Aging (CLSA), including 8,299 participants.^24^ This dataset contained summary data for 1,400 MBs, including 1,091 blood metabolites and 309 metabolite ratios. All cited data sources obtained informed consent and ethical approval from participants; therefore, no additional consents or approvals were required.

For this study, SNPs highly correlated with CIPs were used as IVs. Recent studies have identified a limited number of SNPs that are strongly associated with immune cell phenotypes and blood metabolite levels using a genome-wide significance threshold of p < 5 × 10^−8^.^25^, ^26^ Building on prior research, we adopted a less stringent threshold (p < 1 × 10^−5^) to ensure sufficient SNPs.^26^ To ensure that the selected IVs were conditionally independent, we applied a stringent Linkage Disequilibrium (LD) threshold of *r*^2^ < 0.001, with a clumping window of 10,000 kb.^22^ This criterion minimizes redundancy among SNPs and prevents the inclusion of highly correlated variants, ensuring that each IV provides independent information for the MR analysis. For reverse MR analysis, SNPs related to AR were selected using a threshold of p < 5 × 10^−8^, and LD was addressed similarly (*r*^2^ > 0.001 and clump window < 10,000 kb).^22^ Palindromic SNPs, which display identical alleles on both forward and reverse strands, were omitted to avoid strand ambiguity.^27^ Weak IVs were excluded by calculating the F-statistic using the formula F = R^2^(n-k-1)/*k*(1-R^2^), where *R*^2^ represents the proportion of variance in the exposure explained by the genetic instruments, n is the sample size, and *k* is the number of instruments. SNPs with F < 10 were removed to mitigate weak instrument bias.^28^ The specific flow is illustrated in Fig. 1A.

### Mediation analysis

It remains unclear whether the MBs produced by CIPs after metabolic processing in the human body affect AR. Therefore, we included these MBs as mediators in the analysis. Initially, we conducted a bidirectional MR analysis to assess the potential causal relationship between SNPs associated with CIPs and AR, determining the overall effect (c). Subsequently, a two-step MR analysis was performed to explore the mediating role of MBs. The first step examined the influence of CIPs on MBs, and the second step evaluated the impact of MBs on AR. The indirect effect (a × b) was calculated as the product of these estimates, while the direct effect (c') was derived by subtracting the indirect effect from the overall effect (c − a × b).^29^

### MR analyses

The primary results were obtained using Inverse-Variance Weighted (IVW) analysis, with statistical significance defined as p < 0.05.^30^ The IVW method assumes that all genetic variants are valid instrumental variables, though this assumption may not always hold in practice.^31^ To address this limitation, we employed additional statistical methods, including the MR-Egger method, weighted median method, simple model method, and weighted model method.^22^ These methods allow for consistent estimation of causal effects without requiring all genetic variants to be valid instruments.

### Sensitivity analysis

Following the MR analysis, we conducted rigorous quality control procedures to validate the established causal relationships. These included assessments of genetic pleiotropy and heterogeneity, as well as the implementation of a “leave-one-out” technique.^14^ Heterogeneity was assessed using Cochran’s *Q* test, with a significance threshold set at p < 0.05.^32^ An IVW random-effects model was used in scenarios with heterogeneity, while a fixed-effects model was applied in other contexts.^33^ To further ensure robustness, the MR-Egger and MRPRESSO methods were employed to identify horizontal pleiotropy.^22^, ^34^, ^35^, ^36^ MR-Egger regression is used to detect horizontal pleiotropy, where genetic variants influence the outcome through pathways other than exposure.^36^ The method tests if the intercept of the regression differs from zero, indicating the presence of pleiotropy. A non-zero intercept suggests potential bias in the causal estimates from the IVW method. The key assumption is that pleiotropic effects are uncorrelated with the exposure's effect. If the intercept is zero (or close to zero), pleiotropy is unlikely, and the IVW results are considered reliable.^22^, ^34^, ^36^ MRPRESSO identifies and removes outlier SNPs that could distort the causal estimates.^22^ Outliers are detected based on their residuals in a regression model, and their removal helps adjust for pleiotropic effects. This method assumes that outliers represent genetic variants with pleiotropic effects, which violate the instrumental variable assumptions. Adjusted results are provided to reflect the impact of removing these outliers.^22^, ^35^ Additionally, a leave-one-out analysis was performed to exclude SNPs that had disproportionate effects on the overall causal estimates.

## Results

### SNPs of CIPs and MBs

Based on the IV selection criteria, a total of 2,945 SNPs were selected as IVs for 91 CIPs, and 34,843 SNPs were selected as IVs for 1,400 MBs (Supplementary Tables S1,S2).

### Causal effects between CIPs and AR

**Fig. 2:**
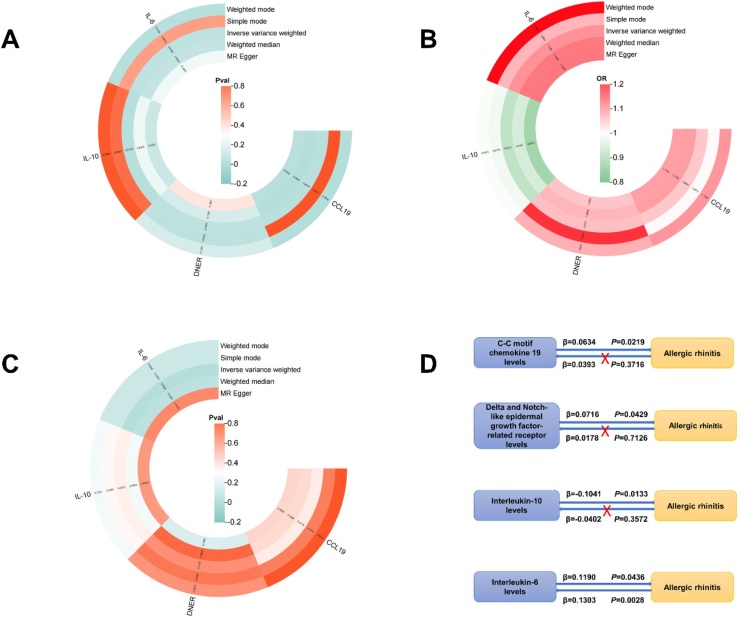
Bidirectional MR analysis between CIPs and AR. (A) The circular heatmap of p-value with CIPs as the exposure and AR as the outcome. (B) The circular heatmap of OR with CIPs as the exposure and AR as the outcome. (C) The circular heatmap of p-value with AR as exposure and CIPs as outcome. (D) Schematic representation of the results of bidirectional MR analysis. CCL19, C-C motif Chemokine-19 levels; DNER, Notch-like Epidermal growth factor-related Receptor levels; IL-10, Interleukin-10 levels; IL-6, Interleukin-6 levels; OR, Odds Ratio; CI, Confidence Interval.

Using the IVW method, significant associations were found between genetically predicted elevated levels of C-C motif Chemokine-19 (CCL19) (OR = 1.065, 95% CI 1.009‒1.125, p = 0.0219), notch-like epidermal growth factor-related receptor (DNER) (OR = 1.074, 95% CI 1.002‒1.151, p = 0.0429), and Interleukin-6 (IL-6) (OR = 1.126, 95% CI 1.003‒1.264, p = 0.0436) with an increased risk of AR. Conversely, Interleukin-10 (IL-10) (OR = 0.901, 95% CI 0.830‒0.979, p = 0.0133) was found to have protective effects against AR (Supplementary Table S3). The causal relationships were illustrated through circular heatmaps (Fig. 2A‒B). Further insights were provided by scatter plots, forest plots, funnel plots, and leave-one-out plots, as shown in Supplementary Figure S1. Sensitivity analyses confirmed the robustness of these results, with no significant evidence of pleiotropy or heterogeneity (Supplementary Table S4). Additional data are available in Supplementary Table S3. In the reverse MR analysis, IL-6 demonstrated a reverse causal relationship with AR, while no significant associations were found for other CIPs (Fig. 2C–D). Then, we performed the Steiger test (Correct causal direction = TRUE, p = 7.84 × 10^−9^), which indicated that the causal relationship is primarily from IL-6 to AR, rather than the reverse, supporting IL-6’s role as a risk factor.

### Causal effects of CIPs on MBs

To estimate the β₁ effect (a), we analyzed the causal effect of CIPs on MBs. The two-sample MR analysis revealed significant causal effects of CIPs on several MBs. Among the CIPs associated with AR, CCL19 was a risk factor for the Adenosine 5'-Monophosphate (AMP) to urate ratio (OR = 1.109, 95% CI 1.012‒1.215, p = 0.0269), and DNER for N-acetylputrescine (OR = 1.114, 95% CI 1.018‒1.219, p = 0.0186). In contrast, IL-10 was protective against plasma lactate (OR = 0.890, 95% CI 0.819‒0.966, p = 0.0056) (Supplementary Table S5). These findings are visualized in Supplementary Figure S3 and were confirmed by sensitivity analyses (Supplementary Table S6). Detailed results are presented in Supplementary Table S5.

### Causal effects of MBs on AR

**Fig. 3:**
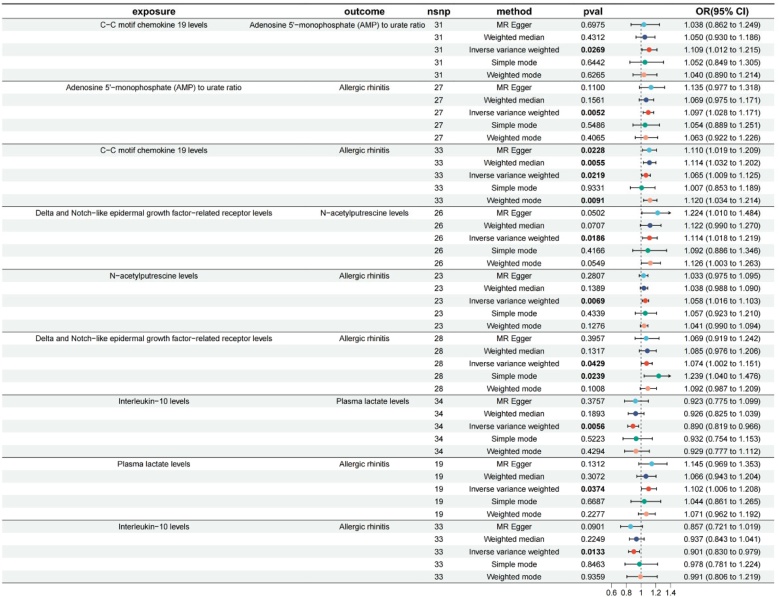
Forest plot to visualize the causal effects of MBs with CIPs and AR.

To estimate the β₂ effect (b), we analyzed the causal effect of MBs on AR risk. The analysis revealed significant causal effects of specific MBs on AR risk. Elevated levels of N-acetylputrescine (OR = 1.058, 95% CI 1.016‒1.103, p = 0.0069), plasma lactate (OR = 1.102, 95% CI 1.006‒1.208, p = 0.0374), and the AMP to urate ratio (OR = 1.097, 95% CI 1.028‒1.171, p = 0.0052) were found to increase the risk of AR (Fig. 3). These results were robust in sensitivity analyses (Supplementary Table S7), and visualizations are provided in Supplementary Figure S3. Additional details are available in Supplementary Table S8.

### Mediation of the association between CIPs and AR by MBs

After identifying the effects of CIPs on mediators and the significant mediators affecting AR, the mediated effect proportion and direct effect were calculated. IL-10’s effect was mediated by plasma lactate (mediated proportion = 10.9%, mediated effect = −0.0113), CCL19’s effect was mediated by the AMP to urate ratio (15.1%, mediated effect = 0.0096), and DNER’s effect was mediated by N-acetylputrescine (8.55%, mediated effect = 0.0061) (Supplementary Table S9). These findings indicate that MBs play a significant role in the pathway from CIPs to AR.

## Discussion

As indicated by the results, our MR analysis identified four CIPs with significant associations: CCL19, DNER, and IL-6 were found to be risk factors, while IL-10 was a protective factor. Furthermore, our MR analysis suggests that certain MBs play a mediating role in AR development. Elevations in N-acetylputrescine, plasma lactate, and the AMP-to-urate ratio were associated with increased AR risk. These findings, when considered alongside prior research on these small molecules in other allergic diseases, particularly AR, provide valuable insights into potential therapeutic targets.

CCL19, a member of the C-C chemokine family, has been implicated in various immune and inflammatory processes.^37^ Previous studies have demonstrated that CCL19 plays a crucial role in allergic diseases, including asthma, allergic conjunctivitis, and AR.^38^, ^39^ Our findings suggest a potential causal relationship between genetic variants associated with increased CCL19 expression and an elevated risk of AR. Studies have shown that CCL19 recruits immune cells and facilitates their migration to lymphoid organs by binding to its receptor, C-C Chemokine Receptor-7 (CCR7), expressed on immune cells such as dendritic cells and T-cells.^40^, ^41^ This regulation of immune cell trafficking and homing underscores CCL19’s role in modulating immune responses and maintaining immune homeostasis. Polymorphisms in the CCL19 gene have been associated with altered CCL19 levels in the nasal mucosa and increased susceptibility to AR. In our study, we found that CCL19-mediated risk for AR was further influenced by purine metabolism, specifically through the AMP-to-urate ratio.^42^ Previous research has linked changes in Prostaglandin E2 (PGE2) levels to purine metabolism and shown that PGE2 induces CCR7 expression on monocytes, enabling them to respond to CCL19 and migrate.^43^, ^44^ Thus, we speculate that PGE2 may play a role in the pathogenesis of AR mediated by CCL19 through the AMP-to-urate ratio, likely related to PGE2-mediated inflammation. These findings indicate that dysregulation of CCL19 expression contributes to AR pathogenesis.

DNER, a Delta/Notch-like epidermal growth factor-related receptor, is a transmembrane protein involved in various biological processes, particularly neural development and synaptic plasticity.^45^ Although DNER has been identified as a risk factor for AR in our MR analysis, there is limited evidence supporting its role in allergic diseases. A proteomic analysis mentioned DNER as a biomarker for assessing asthma control,^46^ and given that AR is a major risk factor for uncontrolled asthma,^3^ this might suggest DNER’s involvement in AR as well. However, the underlying mechanisms remain unclear. As a proposed ligand for NOTCH, DNER may contribute to NOTCH-mediated pro-inflammatory effects,^47^ but further research is needed to confirm this hypothesis. In this analysis, we found that DNER increased the risk of AR by promoting N-acetylputrescine, a metabolite involved in polyamine metabolism. Previous studies have linked elevated plasma levels of N-acetylputrescine with ulcerative colitis,^48^ an inflammatory disease, but its specific role in AR remains unclear. The lack of direct observational evidence on the DNER-AR relationship underscores the need for further functional studies and prospective epidemiological research to validate our MR findings.

Previous studies have demonstrated that inflammatory cytokines play a crucial role in immune regulation and inflammatory responses.^49^, ^50^ IL-6 is a pleiotropic cytokine with well-established pro-inflammatory properties,^50^ and accumulating evidence suggests its involvement in the pathogenesis of allergic diseases. IL-6 exacerbates allergic reactions by inducing the production of Th2-type cytokines.^50^ Tao et al. demonstrated that blocking Th2 signaling can mitigate the inflammatory response and alleviate clinical symptoms for allergic conjunctivitis.^17^ Our MR analysis provides genetic evidence supporting IL-6 as a risk factor for AR, where higher genetically predicted IL-6 levels were significantly associated with an increased risk of AR. Steiger directionality testing confirmed that IL-6 influences AR rather than AR influencing IL-6 levels, further reinforcing its role as an upstream mediator of allergic inflammation. However, no relevant metabolic biomarkers were identified as mediators of IL-6’s pro-inflammatory effects, suggesting that IL-6 may contribute to AR through direct immune modulation rather than via metabolic pathways.

IL-10 is well-known for its anti-inflammatory properties, particularly its ability to suppress pro-inflammatory cytokines. By inhibiting the production of Th2 cytokines such as IL-4, IL-5, and IL-13, which are central to AR pathogenesis, IL-10 can reduce the risk and severity of AR.^51^ IL-10 also promotes the differentiation and function of regulatory T-cells (Tregs), which are essential for maintaining immune tolerance and preventing excessive immune responses.^52^ Elevated IL-10 levels may enhance Treg activity, further mitigating AR development. However, while observational studies have suggested an association between IL-10 levels and reduced AR risk, they are inherently limited by residual confounding and reverse causality. For instance, individuals with severe AR may have altered cytokine expression due to the disease itself rather than IL-10 being causally protective. By leveraging genetic variants as IVs, MR analysis provides stronger causal evidence, indicating that IL-10 plays a protective role in AR pathogenesis.

Beyond its role in immune regulation, IL-10 also influences cellular metabolism.^53^, ^54^, ^55^ Lactate, a key metabolite generated during anaerobic glycolysis, often reflects cellular metabolism and oxygenation status.^53^ Significant changes in plasma lactate levels occur during oxidative stress, immune reactions, and other pathological conditions.^54^ Our findings revealed that IL-10 may protect against AR by reducing plasma lactate levels, providing a mechanistic link between immune regulation and metabolic alterations in allergic diseases. Prior studies have demonstrated that IL-10 inhibits glucose uptake and glycolysis, processes that are highly activated during immune responses.^55^ Collectively, these findings indicate that IL-10 not only exerts direct anti-inflammatory effects in AR but may also modulate metabolic pathways to mitigate disease progression, highlighting IL-10-mediated metabolic regulation as a potential therapeutic target in allergic diseases.

By employing MR analysis, we minimized the influence of confounding factors and enhanced the robustness of our findings by leveraging GWAS data.^22^, ^31^ Compared to traditional observational studies, MR reduces biases from reverse causality and unmeasured confounding, thereby improving causal inference. However, several limitations should be acknowledged.^19^ SNP selection constraints may introduce bias, as genetic variants associated with MBs and CIPs exhibit variability across different populations and datasets, potentially affecting the validity and reproducibility of our findings.^22^, ^28^ Additionally, while MR is designed to minimize confounding, it does not eliminate all sources of bias, particularly horizontal pleiotropy, where SNPs influence the outcome through pathways unrelated to the exposure.^22^, ^34^ Although we employed MR-Egger and MRPRESSO to detect and correct for pleiotropy, residual pleiotropy remains a possibility. Furthermore, unmeasured confounders, such as environmental exposures and gene-environment interactions, may still impact our results despite the genetic-based approach.^22^ Another limitation is that our MR estimates reflect lifetime genetic predispositions to exposure, which may not directly translate into the effects of short-term clinical or behavioral interventions, a crucial distinction when interpreting MR findings in the context of potential therapeutic strategies.^22^, ^30^ Lastly, multiple hypothesis testing increases the risk of false-positive findings, and our study did not adjust for this using the False Discovery Rate (FDR), highlighting the need for future studies to incorporate strict multiple-testing corrections and replicate findings in independent cohorts to ensure the robustness of causal inferences.^56^ Despite these limitations, our study provides stronger causal evidence than traditional observational studies, offering insights into potential biological pathways for AR and informing future research directions.

## Conclusion

In conclusion, our study utilizing MR provides preliminary evidence of the impact of CIPs and MBs on the development of AR. These findings hold clinical significance in enhancing our understanding of AR and may guide future drug development and treatment strategies. However, further validation studies are needed to confirm these findings and broaden their applicability across diverse populations.

## ORCID

Xiang Cao: 0009-0000-8727-1716.

Boyang Zhang: 0009-0005-1860-4392.

Wei Wang: 0009-0002-7391-3866.

Zijiao Xu: 0009-0002-2705-6568.

Zhixin Jiang: 0000-0002-8089-9268.

## CRediT authorship contribution statement

ZJ were responsible for the conception and design of the study. XC, BZ, WW and ZX were responsible for the acquisition and analysis of data. XC and BZ wrote the paper. ZJ reviewed the paper. All authors read and approved the final manuscript for submission. XC and BZ contributed equally to this work.

## Ethical approval

This study utilized publicly accessible GWAS summary statistics for secondary analysis, with all individual GWAS studies receiving prior approval from their respective ethics committees.

## Funding

This work was supported by the 10.13039/501100001809National Natural Science Foundation of China (32200684), the Tianjin Health Research Project (TJWJ2022QN078), the Tianjin Key Medical Discipline (Specialty) Construction Project (TJYXZDXK-016A).

## Data availability

The data are available on reasonable request.

## Declaration of competing interest

The authors declare no conflicts of interest.
