# Supplementary material for: Metabolic biomarkers mediate allergic rhinitis via circulating inflammatory proteins: Evidence from a Mendelian randomization study
Source: Braz J Otorhinolaryngol. 2025 Jun 25;91(5):101658. doi: 10.1016/j.bjorl.2025.101658 (PMC12246841; doi:10.1016/j.bjorl.2025.101658)

**BJORL-D-24-00535_ Supplementary Materials**

**Supplementary Table S1** Detailed information on the included SNPs of CIPs.

**Supplementary Table S2** Detailed information on the included SNPs of metabolites.

**Supplementary Table S3** MR analysis of the CIPs on AR.

**Supplementary Table S4**

**Supplementary Table S5** MR analysis of the CIPs on metabolites.

**Supplementary Table S6.1** Heterogeneity analysis of the CIPs on metabolites.

**Supplementary Table S6.2** Pleiotropy analysis of the CIPs on metabolites.

**Supplementary Table S7.1** Heterogeneity analysis of the metabolites on AR.

**Supplementary Table S8** MR analysis of metabolites on AR.

**Supplementary Table S9** Mediated effect analysis.

**Supplementary Figure S1** Visualization of MR analysis between CIPs and AR, including scatter plots, forest plots, funnel plots, and leave-one-out plots of single SNPs. (A‒D) The causal effect of CCL19 associated SNPs on AR. (E‒H) The causal effect of DNER associated SNPs on AR. (I‒L) The causal effect of IL-10 associated SNPs on AR. (M‒P) The causal effect of IL-6 associated SNPs on AR. CCL19, C-C motif Chemokine-19 levels; DNER, Notch-like Epidermal growth factor-related Receptor levels; IL-10, Interleukin-10 levels; IL-6, Interleukin-6 levels.


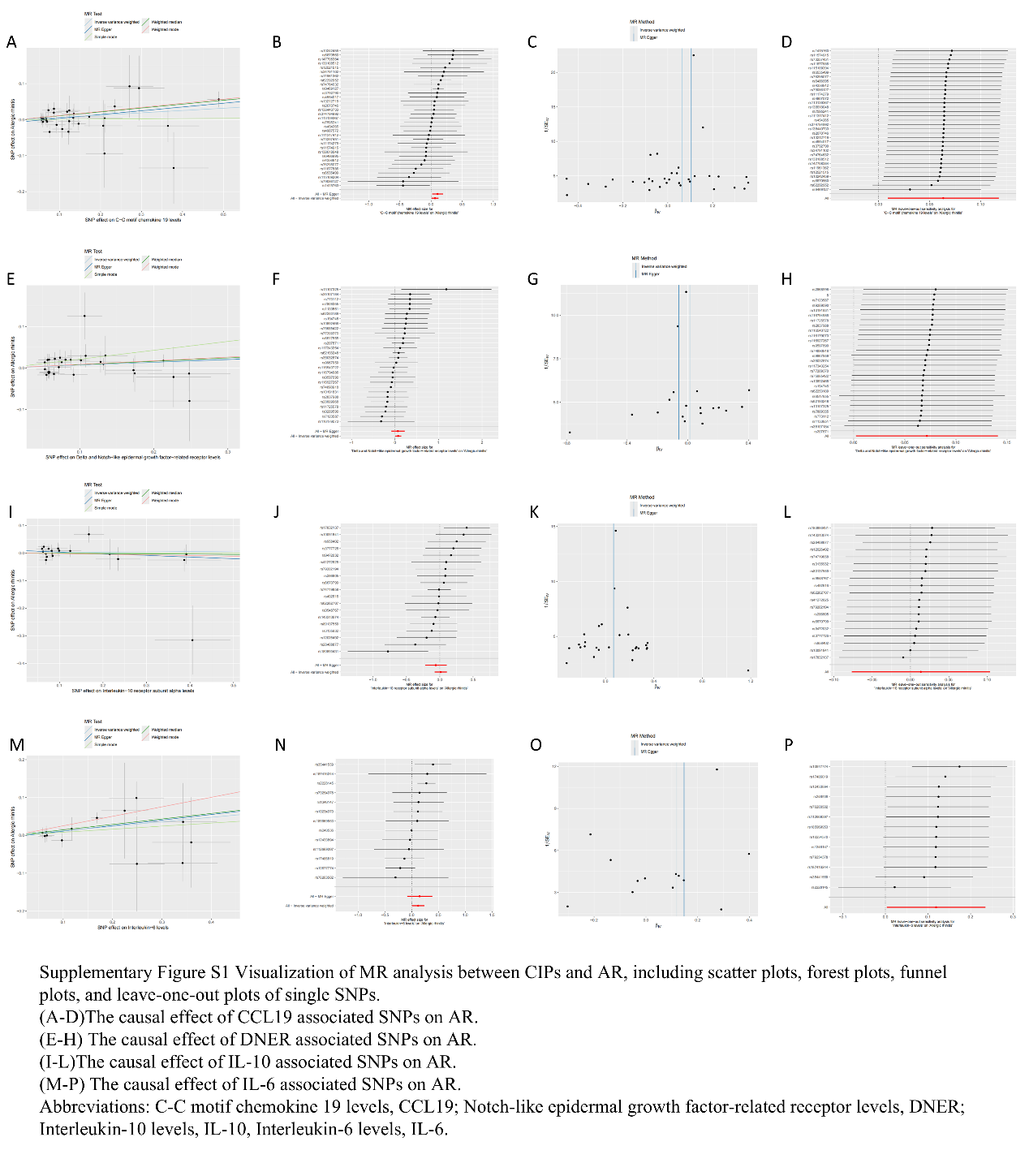


**Supplementary Figure S2** Visualization of MR analysis between CIPs and AR, including scatter plots, forest plots, funnel plots, and leave-one-out plots of single SNPs. (A‒D) The causal effect of CCL19 associated SNPs on adenosine 5’-monophosphate (AMP) to urate ratio. (E‒H) The causal effect of DNER associated SNPs on N-acetylputrescine. (I‒L) The causal effect of IL-10 associated SNPs on plasma lactate. Metabolite ID: GCST90200976, adenosine 5’-monophosphate (AMP) to urate ratio; GCST90200351, N-acetylputrescine; GCST90200408, plasma lactate. CCL19, C-C motif Chemokine-19 levels; DNER, Notch-like Epidermal growth factor-related Receptor levels; IL-10, Interleukin-10 levels.


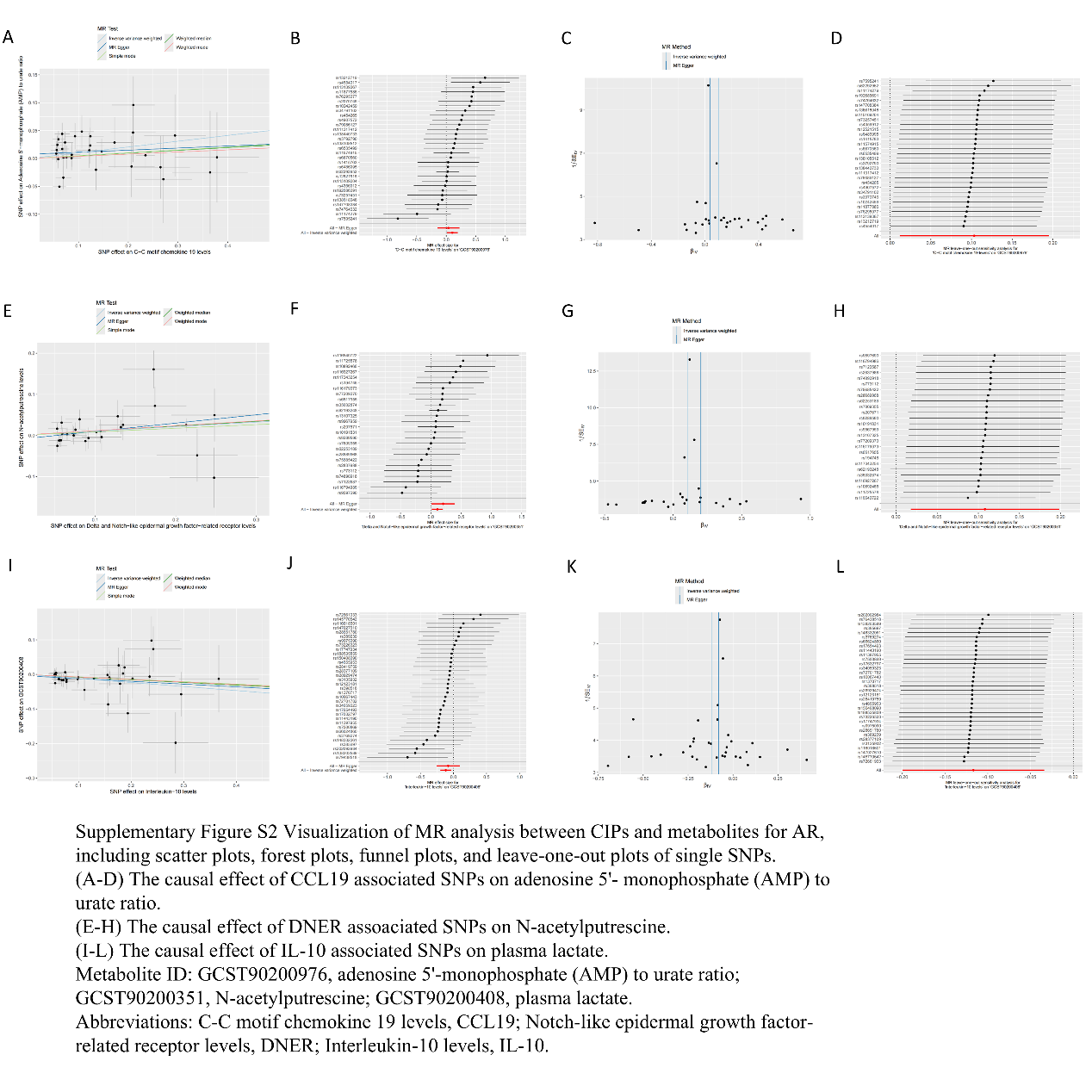


**Supplementary Figure S3** Visualization of MR analysis between metabolites and AR, including scatter plots, forest plots, funnel plots, and leave-one-out plots of single SNPs. (A‒D) The causal effect of N-acetylputrescine associated SNPs on AR. (E‒H) The causal effect of adenosine 5’-monophosphate (AMP) to urate ratio associated SNPs associated SNPs on AR. (I‒L) The causal effect of plasma lactate associated SNPs on AR.


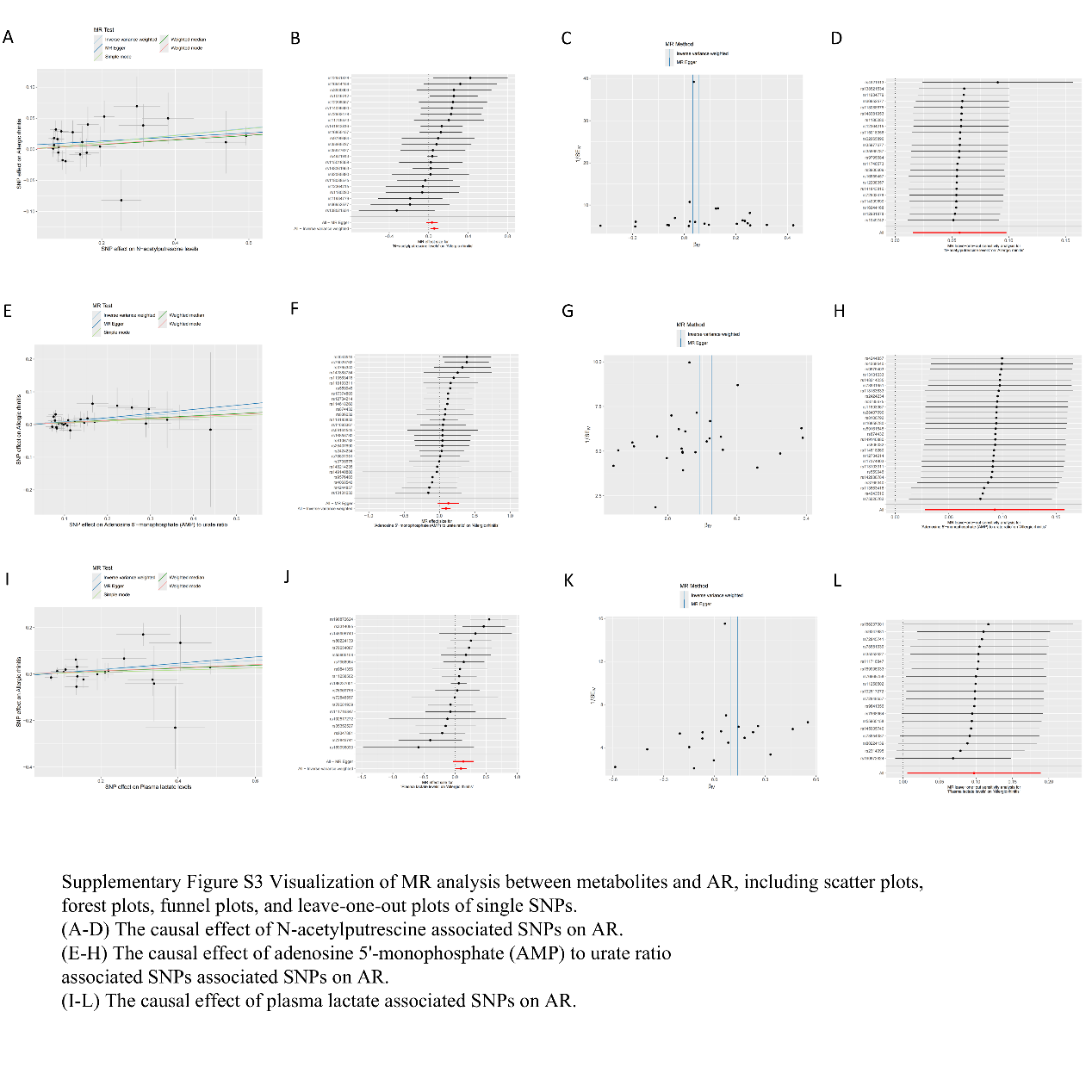

Supplement: Supplementary file 1 [file mmc1.docx]
